# The Role and Mechanism of the Vascular Endothelial Niche in Diseases: A Review

**DOI:** 10.3389/fphys.2022.863265

**Published:** 2022-04-27

**Authors:** Zhiqiang Lei, Xiang Hu, Yaoqi Wu, Longsheng Fu, Songqing Lai, Jing Lin, Xiaobing Li, Yanni Lv

**Affiliations:** ^1^ School of Clinical Medicine, Jiangxi University of Chinese Medicine, Nanchang, China; ^2^ Department of Pharmacy, The First Affiliated Hospital of Nanchang University, Nanchang, China

**Keywords:** vascular endothelial niche, tumor metastasis, blood, chemokine, adhesion molecules, tight junction, inflammatory factor

## Abstract

Vascular endothelial cells, forming the inner wall of the blood vessels, participate in the body’s pathological and physiological processes of immunity, tumors, and infection. In response to an external stimulus or internal pathological changes, vascular endothelial cells can reshape their microenvironment, forming a “niche”. Current research on the vascular endothelial niche is a rapidly growing field in vascular biology. Endothelial niches not only respond to stimulation by external information but are also decisive factors that act on neighboring tissues and circulating cells. Intervention through the vascular niche is meaningful for improving the treatment of several diseases. This review aimed to summarize reported diseases affected by endothelial niches and signal molecular alterations or release within endothelial niches. We look forward to contributing knowledge to increase the understanding the signaling and mechanisms of the vascular endothelial niche in multiple diseases.

## Introduction

The definition of niche originated from the ecological environment, representing the environment and living habits for each species. In recent years, the concept of a “niche” has been introduced into the medical field, especially the field of oncology (Rafii et al., 2016). Metastatic tumors prefer certain tissue locations or niches for their growth and metastasis. The microenvironment can be identified as a ‘niche’ as well. It is believed that the microenvironment, namely, the “niche”, promotes the metastasis of cancer cells to specific nonadjacent organs through the circulatory system. The microenvironmental “niche” is characterized by abnormal glucose metabolism, disturbance of lipid metabolism, abnormal protein signaling molecule secretion, and so on, providing a supportive microenvironment for inflammation, immunosuppression, angiogenesis, vascular permeability, and lymphangiogenesis (Géraud et al., 2014). These characteristics of niche comprise the preferred condition for tumor metastasis. For example, exosomes derived from brain metastatic breast cancer and lung tumors were rich in hyaluronic acid binding protein, which formed the premetastatic ecological niche of the cerebrovascular endothelium and promoted tumor exosomes to brain metastasis (Rodrigues et al., 2019). Colorectal cancer exosomal miR-25-3p promoted niche formation of vascular endothelial to facilitate breast cancer metastasis by inducing vascular permeability and angiogenesis (Zeng et al., 2018a). Interference in the tumor niche might represent a potential therapy to make the niche uninhabitable for tumor cells (Friedman-Levi et al., 2021). In addition to oncology, in recent years, the idea of the niche has attracted attention in central nervous, cardiovascular, lung, inflammatory, and liver diseases.

A niche can be formed by any organ, tissue or cells, among which the role of the vascular endothelial cell niche is the most extensive. Blood vessels are formed by vascular endothelial cells, mural cells, perithelial cells, and other cells that form protective barriers and provide molecular nutrition (Psaltis et al., 2011). Current findings indicate that vascular endothelial cells play a special niche role that expand their original roles. For example, under normal conditions, the vascular endothelial microenvironment is a “harmful environment” for neutrophil migration in the brain tissue. Even if a small amount of harmful molecules stick to the vascular endothelial wall, it would not penetrate the vascular endothelium, resulting in low efficiency of recruitment and migration of harmful molecules (Nikolova et al., 2007). In response to an external stimulus or internal pathological changes, vascular endothelial cells reshape their microenvironment, forming a “niche”, which support and promote harmful cell recruitment to specific tissues from the circulatory system. Thus, the vascular endothelial niche can only be fully interpreted when its specific niche components and their functions are well understood. Therefore, this review explores the changes that occur in the vascular endothelial niche responsible for pathologic changes. As a result, new vascular endothelial intervention molecular therapies could be explored as potential therapies for more diseases.

## Part One: The Conception and Connotation of Vascular Endothelial Niche

In nature, amount species tend to occupy areas with certain characteristics, such as the specific area composed of plant, soil, and germs. Ecologists have developed the concept of a “niche” to delineate the way that organisms adapt to their environment. A “niche” is described as a constantly changing set within environmental factors, which keep a specie long lives in survival (Zhang C. D. et al., 2022). The internal essence for “niche” might be addressed as a physical factor limit for the adaptive environment, determining the extent of its geography or habitat in general, whereas survival within this range depending on competing resources with other species living there (Rubalcaba et al., 2020). The concept of “niche” goes to the heart of ecology--two critical components of “niche”: organisms and environment. When the resources and environmental conditions were changed by organisms, the organisms themselves also underwent the changes (Hoshizaki et al., 2022).

The conception of “niche” is introduced from ecology to medical field. The term “vascular niche” refers to the established microenvironment around blood vessels, in which endothelial cells, hemodynamic mechanical forces, stromal cells, parenchymal cells, extracellular matrix molecules, or pericytes, and smooth muscle cells contribute to the formation of blood vessels. Thus, a mature blood vessel contains several cell populations, all of which could contribute to the formation of vascular niche. The environment of blood vessels affect its differentiation, survival and proliferation, and even exert on the adjacent cells or tissues or circulating cells (Ribatti et al., 2021). Blood vessel endothelium composed of a thin layer of epithelial cells, characteristic with flattened, polygonal, serrated edges. Vascular endothelial cells form the lining of blood vessels and also act as the interface between blood and other vascular walls. Along the entire circulatory system, vascular endothelial cells exist from the heart down to the smallest blood vessels (Ferentinos et al., 2022). Vascular endothelial cells located between plasma and vascular tissues, it could not only complete the metabolism exchange between plasma and tissue fluid, but also synthesize and secrete a variety of biological active substances in order to ensure the normal contraction of blood vessels and maintain the normal flow of blood.

The specific term “vascular endothelial niche” was defined as a microenvironment that is generated by vascular endothelial cells affects the behavior of adjacent cells or the internal circulatory system like blood, circulating cells, etc. Since the vascular vessels composed of variety cellular populations, like hemodynamic mechanical forces, stromal cells, parenchymal cells, extracellular matrix molecules, or pericytes, and smooth muscle cells, there is relatively straightforward interaction between vascular endothelial cells and adjacent cells of vascular vessels. Stromal cells are the cells to assist parenchymal cells to perform organ functions in specific organ. Zhang et al.(2022) demonstrated that circANKRD36 regulated miR-599 and TGF-β signaling pathway to promote endothelial mesenchymal transition in aortic valve stromal cells. In Jackson’ paper (Jackson, 2022), endothelial cells activated channels as transient receptor vanilloid family member 4 (TRPV4) channels, promoting intermediate and small conductance Ca^2+^-activated K^+^ (IKCa and SKCa) to transmit signaling molecules to smooth muscle via intercellular junctions, which controlling the activity of smooth muscle or pericyte contraction (Jackson, 2022). Therefore, endothelial ion channels made a microcirculation and in body balance by participating in cell-cell communication (Jackson, 2022). Macromolecules secreted by cells into the extracellular stroma form a complex network that supported tissue structure, called extracellular matrix molecules, which regulated tissue genesis and cellular physiological activities. Not just adjacent cells, vascular endothelial cells had the functions on hemodynamic mechanical forces. Embryo restricted transcription factor variant 2 (ETV2) was instantaneously reactivated in mature human endothelial cells, forming a perfusion plastic vascular plexus to facilitate organ development and tumorigenesis (Palikuqi et al., 2020).

The term “niche” also own the characteristics of stem cells (Li and Xie, 2005). The term vascular endothelial stem indicated the physical and biochemical microenvironment around blood vessel where endothelial cells, pericytes, and smooth muscle cells organize themselves to form blood vessels and release molecules involved in the recruitment of hematopoietic stem cells, endothelial progenitor cells, and mesenchymal stem cells (Abkowitz et al., 2003; Kopp et al., 2005). Vascular endothelial stem form a reticular network that supports the formation and the interaction within hematopoietic stem cells, endothelial progenitor cells, and mesenchymal stem cells. Transmembrane adhesion glycoproteins and chemokines participated in the migration of hematopoietic stem cells and endothelial progenitor cells from endothelial cells within specific niche in bone marrow (Willert et al., 2003). The vascular endothelial stem niche within bone marrow regulated stem cell mobilization, proliferation, and differentiation by cell-cell communication through the secretion of vascular endothelial growth factor, fibroblast growth factor, interleukins, transforming growth factor, platelet-derived growth factor, and nitric oxide (Pasquier et al., 2020).

## Part Two: Vascular Endothelial Niches and Diseases

**FIGURE 1:**
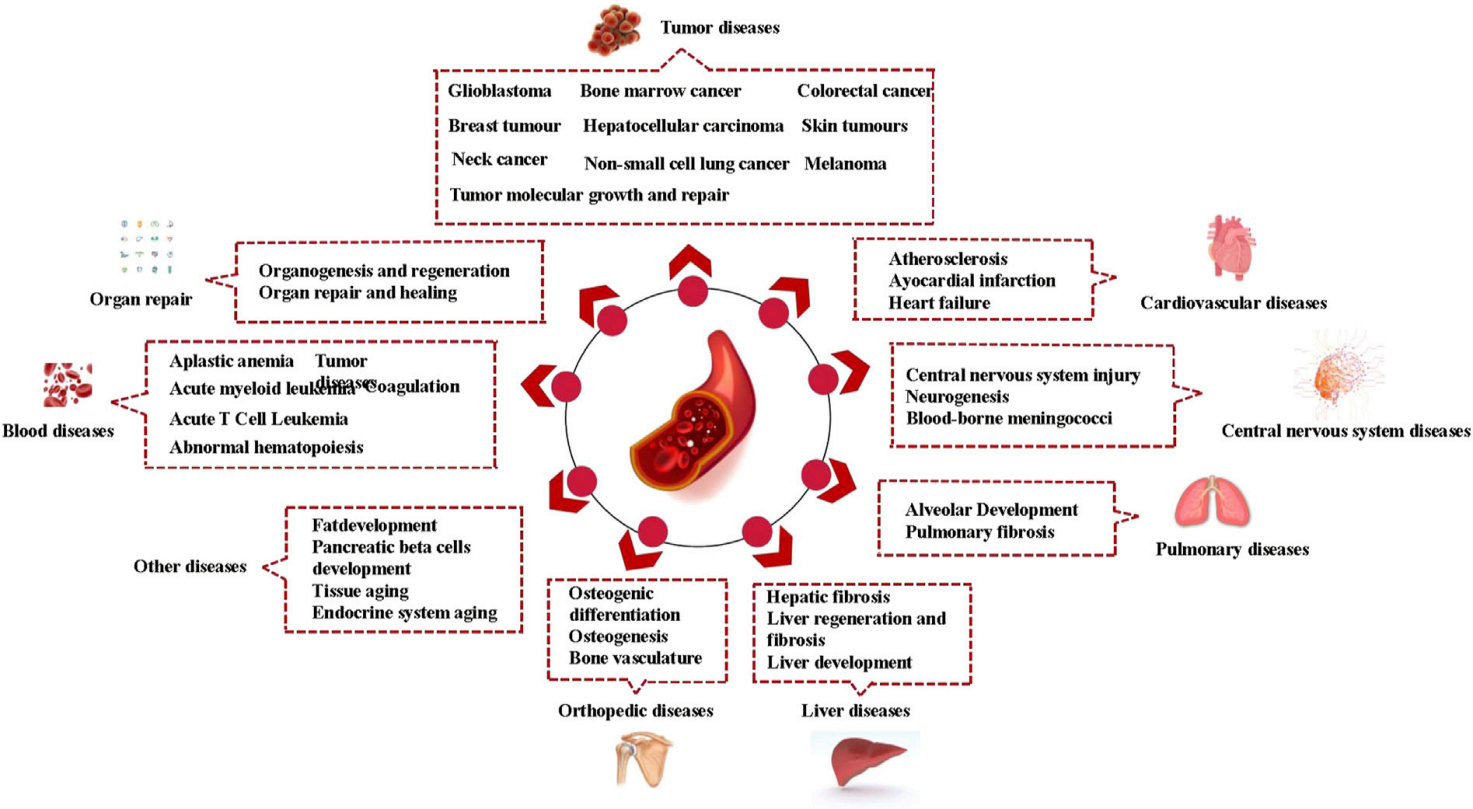
Vascular endothelial niches and diseases.

The role of vascular endothelial niches is quite extensive. Vascular endothelial niches can reshape their microenvironment, playing a role in various diseases. In addition to tumors, which are the most frequently reported niches in the literature, vascular endothelial niches provide a supportive microenvironment for other diseases, such as blood diseases, cardiovascular disease, central nervous diseases, pulmonary pathologies, liver disease, and orthopedic diseases. The changes of vascular endothelial niches in specific diseases could be seen in Table 1; Figure 1.

**TABLE 1:** The changes of vascular endothelial niches in specific diseases.

Diseases	Specific diseases	Changes in vascular endothelial niches
Tumor diseases	Glioblastoma (Sharma and Shiras, 2016; Ho and Shim, 2017).	Secretion of platelet derived growth factor and nitric oxide, and regulation of notch, TGF-β, nitric oxide pathway, etc
	Bone marrow cancer (Ribatti et al., 2014).	Angiogenesis
	Colorectal cancer (Zeng et al., 2018b).	Alternations in tight junction proteins
	Breast tumor (Ghajar et al., 2013).	Secretion of platelet derived growth factor
	Hepatocellular carcinoma (Yu et al., 2010).	Hypoxia condition
	Non-small cell lung cancer (Li et al., 2019).	Release of inflammatory factors
	Skin tumors (Beck et al., 2011).	Secretion of vascular endothelial growth factor
	Neck cancer (Zhang et al., 2014).	Secretion of epidermal growth factor
	Melanoma (Lai et al., 2012).	The formation of channels
	Tumor molecular growth and repair (Butler et al., 2010; Singhal and Augustin, 2020).	Secretion of angiocrine factors
	Bone tumor metastasis (Singh et al., 2019).	Secretion of angiocrine factors
Blood diseases	Aplastic anemia (Wu et al., 2017).	Secretion of vascular endothelial growth factor
	Abnormal hematopoiesis (Hochstetler et al., 2019).	Genetic alternation
	Coagulation (Nguyen et al., 2018).	Blood coagulation
	Acute T cell leukemia (Pitt et al., 2015).	Secretion of chemokines
	Acute myeloid leukemia (Cogle et al., 2014).	Upregulation of CD105
Organ repair	Organogenesis and regeneration (Ribatti et al., 2021).	Angiogenesis
	Organ repair and healing (Rafii et al., 2016).	Secretion of angiocrine factors
Cardiovascular diseases	Atherosclerosis (Poller et al., 2020).	Secretion of chemokines and regulation of notch pathway
	Myocardial infarction (Zhang et al., 2012).	Secretion of vascular endothelial growth factor
	Heart failure (Qian et al., 2008).	Angiogenesis
Central nervous system diseases	Central nervous system injury (Madri, 2009).	Hypoxia condition
	Neurogenesis (Ohab et al., 2006).	Secretion of vascular endothelial growth factor
	Blood-borne meningococci (Capel et al., 2017).	Angiogenesis
Pulmonary diseases	Alveolar Development (Mammoto and Mammoto, 2019).	Secretion of angiocrine factors
	Pulmonary fibrosis (Andersson-Sjöland et al., 2016).	Release of inflammatory factors
Liver diseases	Hepatic fibrosis (Cao et al., 2017).	Genetic alternation
	Liver regeneration and fibrosis (Ding et al., 2014; Shido et al., 2017).	Secretion of angiocrine factors
	Liver development (Géraud et al., 2017)	Genetic alternation
Orthopedic diseases	Osteogenic differentiation (Tsai et al., 2015).	Secretion of angiocrine factors
	Osteogenesis (Owen-Woods and Kusumbe, 2022).	Angiogenesis, hematopoiesis, and osteogenesis
	Bone vasculature (Kumar et al., 2021).	Secretion of platelet derived growth factor, vascular endothelial growth factor, chemokines, inflammatory factors, and angiogenesis, etc.
Other diseases	Fat development (Hussain et al., 2021).	Alternations in tight junction
	Pancreatic beta cells development (Lui, 2014).	Secretion of vascular endothelial growth factor
	Tissue aging (Chen et al., 2021a).	Alternations in tight junction
	Endocrine system aging (Chen et al., 2021b).	Genetic alternation

## Part Three: Changes in Vascular Endothelial Niches

### Alternations in Tight Junction Proteins in Vascular Endothelial Niches

Tight junctions exist between vascular endothelial cells and epithelial cells often present as a continuous belt connecting adjacent cells closely together, forming a natural physical barrier between cells. Tight junctions are categorized as physical barriers to the vascular endothelial niche they surround. Linear shape along the junctions was reduced with less distinct connections in response to external and internal stimulation, with weaker tight junction protein signal intensity and volume (Lv and Fu, 2018). Colorectal cancer cells secreted exosome miR-25-3p, which targeted vascular endothelial growth factor (VEGF) receptor 2, zonula occludens 1, occludin, and claudin five on endothelial cells to induce vascular permeability and angiogenesis, forming a premetastatic niche for colorectal cancer metastasis (Zeng et al., 2018a). Additionally, in Lyl1^−/−^ mice, adipose stem cell vascular niche impairment led to decreased pericyte coverage with loss of vascular endothelial (VE)-cadherin and zonula occludens 1, resulting in premature adipose stem cell vascular niche depletion (Hussain et al., 2021).

### Release of Adhesion Molecules From Vascular Endothelial Niches

Factors expressed by endothelial cells mediate the effects of vascular endothelial niches. Cellular adhesion molecules mediate contact and binding between cells and the extracellular matrix. Adhesion molecule function results in cell-to-cell adhesion, cell-to-matrix adhesion, or cell-to-matrix adhesion by virtue of receptor–ligand binding. Adhesion molecules expressed within the vascular endothelial niche are involved in defense functions, inflammation, and tumor metastasis (Cheng et al., 2021). Adhesion molecules are divided into the integrin family, selectin family, immunoglobulin superfamily, and cadherin family according to their structural characteristics. In addition, some adhesion molecules have not yet been classified. Cadherin, selectin, and other adhesion families have been primarily reported to be involved in vascular endothelial niches (Cheng et al., 2021).

Cadherins comprise a family of calcium-dependent adhesion molecules. Endothelial cadherin is a major protein involved in vascular remodeling and vascular integrity. Expression of various adhesion molecules at high levels on the endothelium during inflammation established a premetastatic niche that lead to the recruitment of bone marrow precursors (Grigoryeva et al., 2020). Sphingosine-1-phosphate induced key adhesion proteins of VE-cadherin in pericytes that offset the drastic increase in vascular permeability, highlighting the importance of pericyte-endothelial interactions for vascular stabilization (AbdelRahman et al., 2021). Activation of high p53 expression by VE-cadherin in vascular endothelial cells induced a reduction in the number of perivascular mesenchymal stromal cells around hematopoietic stem cells, promoting hematopoietic stem cell aging and transformation (Si et al., 2018). Brain endothelial cells directly interacted with matriptase in the neural stem cell vascular niche to induce endothelial signaling that is sensitive to cholera toxin (Tung and Lee, 2017). Members of the selectin family recognized and selectively bound specific glycosylates, primarily mediating recognition and adhesion between leukocytes and vascular endothelial cells. The selectin family consisted of L-selectin, P-selectin, and E-selectin, which were mostly expressed on the surface of leukocytes, endothelial cells, and some tumor cells. Combination therapy with the selectin inhibitor GMI-1271 and imatinib prolonged survival in chronic myelocytic leukemia mice by reducing the contact time between leukemia cells and bone marrow endothelial cells (Godavarthy et al., 2020). Blockade of the adhesion molecule E-selectin, which was expressed exclusively by bone marrow endothelial cells, inhibited hematopoietic stem cell proliferation, self-renewal, and chemoresistance (Winkler et al., 2012). In addition to classic adhesion molecules, other adhesion molecules, such as junctional adhesion molecules, mediated endothelial progenitor cells during tube formation in the perivascular niche to reduce their adhesion to the tumor endothelium *in vivo* (Czabanka et al., 2020).

### Secretion of Chemokines From Vascular Endothelial Niches

The critical function of chemokines is to manage the migration of hemameba to their specific position during equilibrium process when the process goes disease disorders. Chemokines directionally induce chemotaxis of nearby reactive cells through promoting proliferation of vascular endothelial cells and angiogenesis (Li et al., 2022). Vascular endothelial cells, chemokine (C-X-C motif) ligand 12 (CXCL12)-rich reticular cells, and mesenchymal stromal cell-regulated chemokines, cytokines, and cell surface adhesion molecules have been identified as microenvironmental cells in neoplastic hematopoiesis (Kaushansky and Zhan, 2018). CXCL12, localized within endothelial cells in the spleen niche, contributed to attracting differentiating hematopoietic cells to facilitate erythroblastic accumulation (Miwa et al., 2013). CXCLL2 deletion from the vascular endothelial vascular niche in T cell acute lymphoblastic leukemia impeded tumor growth (Pitt et al., 2015). The chemokine CXCL6 promoted angiogenesis to remodel the arteriolar niche in acute myeloid leukemia (Li et al., 2021).

### Vascular Endothelial Niches Facilitate Angiogenesis

a) Vascular endothelial cells can form channels in the blood vessel niche to facilitate invasion of molecules into specific tissues or organs. Under normal circumstances, vascular endothelium has its specific morphology and process unabnormal hyperplasia (Tracy et al., 2022). The research has uncovered that when body undergone disruption with tumor or inflammation, vascular endothelial cells proliferated to form a new vascular channel morphology. Endothelial cells in contact with glioblastoma stem cells form channels in the vessel niche to facilitate macrophage invasion into glioblastoma (Schiffer et al., 2018). RNAi-mediated attenuation of CD133 weakens CD144 (VE-cadherin) (+) melanoma cells, resulting in the formation of vessel-like channels and comprising the driving force for melanoma tumor growth (Lai et al., 2012).
b) Platelet derived growth factor (PDGF) is an alkaline protein stored in platelet α particles under normal physiological condition, while released and activated by disintegrating platelets during blood coagulation. It has the biological activity of stimulating chemotaxis and growth of specific cells (Luo et al., 2022). When suffered a severe injury of tissues, macrophage, vascular smooth muscle cells, fibroblasts, endothelial cells, or embryonic stem cells could also synthesis and release PDGF, exerting the function of chemotactic activity, division, phosphate enzyme activation, or prostaglandin metabolism. In a strong adhesion state, endothelial derived thrombospondin 1 gave rise to integrin aggregation and ligand integrin affinity modification. Endothelial derived thrombospondin 1 induced the activation of transforming growth factor β1, and periostin sustained a dormant niche for breast cancer cell quiescence (Ghajar et al., 2013). Thrombospondin 1 combound with hepatocyte growth factor, and laminins derived from islet endothelial cells played a critical role in the vascular endothelial niche in maintaining β-cell function and growth (Olerud et al., 2009).
c) VEGF is a highly specific growth factor involved in promoting vascular permeability, extracellular matrix degeneration, vascular endothelial cell migration, proliferation, and vascular formation (Lai et al., 2022). Given its critical role in mediating angiogenesis, VEGF is involved in the pathogenesis and progression of many angiogenesis dependent diseases, including cancer, inflammatory diseases, and diabetic retinopathy. Inhibition of VEGF stem cells residing in vascular endothelial niches might effectively prolong the survival period in ependymoma cancer (Nambirajan et al., 2014). Tumor-derived VEGF created a perivascular niche for stimulating cancer stemness and renewal through angiogenesis in a paracrine manner (Beck et al., 2011). VEGF activated endothelial cells to create the vascular niche, which enabled leukemic cells to proliferate at a higher rate and increased leukemic adherence to endothelial cells (Poulos et al., 2014). Angiogenesis and the expression of Akt-mediated angiogenic cytokines in R2 porcine hearts induce niches beneficial to cardiac repair (Zhang et al., 2012). VEGF influenced the vascular niche by regulating angiogenic factors that promoted the development of pancreatic beta cells (Lui, 2014). VEGF- and brain-derived neurotrophic factor mediated crosstalk between neural stem cells and endothelial cells to mold the neurovascular niche causing powerful blood vessel formation and maintenance (Li et al., 2006). Helium preconditioning promoted angiogenesis by elevating the mRNA and protein expression of VEGF and angiopoietin 1 to improve the focal neurovascular niche in a neonatal rat hypoxia/ischemia brain injury model (Li et al., 2016). Other growth factors were more involved in central nervous system diseases. Growth differentiation factor 11 enhanced neurogenesis, maintaining the integrity of the cerebrovascular niche in response to the changes that occurred during aging (Katsimpardi et al., 2014). The interaction between neurogenesis and angiogenesis was related to angiogenic matrix derived factor 1 and angiopoietin 1, which promoted neuroblast migration and behavioral recovery after stroke (Ohab et al., 2006).

### Inflammation and Oxidative Stress on Vascular Endothelial Niches

Dysfunctional vascular endothelial cells could promote the aggregation of inflammatory factors (Yamagata, 2019), while endothelial oxidative stress could also cause inflammatory responses to vascular endothelial cells (Marchio et al., 2019), the inflammatory factors and oxidative stress reinforcing each other. The protein kinase CK2 was involved in the perivascular resistant niche stimulation under ionizing radiation, while secreting the cytokines interleukin 8 (IL-8) and IL-6, leading to resistance to radiation in non-small cell lung cancer cells (Li et al., 2019). Endothelial cell derived IL-6 activated the IL-6 receptor and signal transducer and activator of transcription 3, promoting self-renewal of dental pulp stem cells (Oh et al., 2020). A three dimensional model of endothelial cells and cancer stem like cells generated the conditional niche through elevated levels of the IL-8 and IL-8 homologous receptors CXCR1/2, which enhanced the migration, growth, and dryness characteristics of cancer stem-like cells (Infanger et al., 2013). Extracellular proteins from endothelial cells, such as IL-23, IL-17ɑ, dipeptidyl peptidase-4, and recombinant cystatin 3, organized the prometastatic molecular response in regeneration potentiated melanoma (Prakash et al., 2019). Pericytes could increase adenosine, nitric oxide, IL-10, TGF-β1 (Transforming growth factor beta 1), and MHC-II (Major histocompatibility complex-II) levels, which participated in melanoma cell extravasation (Caporarello et al., 2019). The reactive oxygen species-induced endothelial niche might play an important role in the development of pulmonary fibrosis via the regulation of pericytes and Wnt signaling (Andersson-Sjöland et al., 2016). High nitric oxide activity was observed in the tumor vascular endothelium adjacent to perivascular glioma cells and promoted stem-like characteristics in glioma cells (Charles et al., 2010).

### The Hypoxic Niche of Vascular Endothelial Cells

Several hypoxia genes stabilize the internal environment of cells under hypoxia conditions to adapt to hypoxia condition (Jing et al., 2022), while they are involved in many physiological and pathological environments, such as placental development, tumor development, and metastasis. Hypoxia directly damages endothelial cells by destroying the cytoskeleton and intercellular connections, as well as disrupting metabolic and synthetic function, increasing the permeability of endothelial cells. The hypoxic niche of vascular endothelial cells promoted stemness maintenance and tumor propagation in cancer stem cells (Turpin et al., 2015). HIF-1 alpha (Hypoxia inducible factor 1 subunit alpha) mediated responses to hypoxia in central nervous system neurovascular niches inducing several signaling molecules, including BDNF (Brain derived neurotrophic factor), VEGF, and stromal cell-derived factor 1, which were involved in orchestrated angiogenesis and neurogenesis (Madri, 2009). Hypoxic and highly angiogenic areas formed a resulting niche of endothelial progenitor cells, while this particular niche might be related to liver cirrhosis (Yu et al., 2010).

### Genetic Alternations in Vascular Endothelial Cells

Several gene alterations in vascular endothelial genes also affect the function of vascular endothelial cells as an ecological niche. Jagged2 (Jag2) was identified as a ligand of Notch receptor in multiple myeloma patient origin specimens. A lack of Jag2 in the perisinusoidal endothelial niche accelerated aging of hematopoietic stem cells (Saçma et al., 2019). JAK2V617F have shown high mutation rate in myeloproliferative disorders. Endothelial cells with the JAK2V617F mutation was an essential component of the hematopoietic vascular niche and was involved in the pathogenesis of myeloproliferative neoplasms (Lin et al., 2018). Endothelial cells with the kinase mutation JAK2V617F exhibited upregulated expression of CXCL12, and stem cell factors promoted clonal expansion in myeloproliferative neoplasms (Zhan et al., 2018b). Among the subtypes of gene KRAS, KRAS G12D is a common submutation, found in colorectal cancer, pancreatic cancer, and non-small cell lung cancer. Specifically, oncogenic KRas G12D mutations in adult endothelial cells significantly increased leukocytes and bone marrow cells in the blood of mice (Hochstetler et al., 2019). Ephrin receptors make up the largest subgroup of the receptor tyrosine kinase family, the protein encoded by ephrin type-B receptor 4 (EPHB4) plays an essential role in vascular development. EphB4 in the forebrain neurogenic niche participated in the process of vascular, molecular and structural remodeling (Colín-Castelán et al., 2011). Hepatocyte growth factor (HGF) was discovered as a substance that stimulated the proliferation of liver cells. HGF knockout mice displayed abnormally upregulated perivascular NOX4 expression near endothelial cell-induced niches, which promoted regeneration of mouse and human parenchymal cells in damaged organs (Cao et al., 2017). GATA Binding Protein 4 (GATA4) is an important transcription factor in the regulation of gene expression. Deletion of the transcription factor GATA4 in liver sinusoidal endothelial niches caused liver hypoplasia, fibrosis, and impaired colonization by hematopoietic progenitor cells through angiocrine factors (Géraud C. et al., 2017). Receptor activity modifying proteins serve as oligomeric modulators for numerous G-protein coupled receptors. Conditioned knockout of mouse endothelial receptor activity modifying protein 2 facilitated pulmonary endothelial cellular deformation and inflammatory infiltration to mediate the formation of premetastatic niches that ultimately promoted tumor metastasis (Tanaka et al., 2016).

### Others Changes in Vascular Endothelial Niches

The platelet reactive protein family is a group of structurally related secreted proteins that are widely distributed in the extracellular matrix of various tissues and inhibit angiogenesis. The β mural PDGF receptor was subsequently reprogrammed into NeuN^+^ local interneurons to hasten neurogenesis (Farahani et al., 2019). Notch signaling activated PDGF receptor-β positive perivascular cells, leading to the expansion of hematopoietic stem cell niches in bone (Kusumbe et al., 2016). Several signaling or bioactive molecules were secreted by endothelial cells in peripheral or local tissues. Capillary endothelial cells supplied paracrine factors, called angiocrine factors, to adjacent cells in the niche to orchestrate these processes. Endothelial cells regulated hematopoietic stem cell maintenance and regeneration of organ-specific stem cells through endothelial cell-derived paracrine factors (Sasine et al., 2017). Additionally, activation of endothelial cells or endoglin indicated a change in the vascular endothelial niche. Endothelial progenitor cells secreted proangiogenic factors to participate in angiogenesis, particularly under ischemic conditions (Yan et al., 2017). Upregulation of CD105 was associated with activated endothelium, affecting vascular tissue associated acute myelocytic leukemia cells and interactively affecting each other (Cogle et al., 2014). Runt-related genes in the vascular niche revealed that the vascular niche regulated the engraftment of acute myeloid leukemia cells in the bone marrow, influencing the overall survival of leukemic mice (Morita et al., 2018). Soluble amyloid precursor protein from endothelial cells triggered vascular niche functions that negatively regulated growth and restricted the number of neural stem cells in the subventricular zone (Sato et al., 2017).

## Discussion

The vascular endothelial niche is a potential decisive and limiting factor for many pathological processes. Vascular endothelial niches have experienced dynamic changes during the psychological process between health and disease condition. In the health condition, vascular endothelial cells maintained the basic function of blood vessels. Although vascular endothelial cells were considered as passive conduits, progress in vascular endothelial niches research now suggested that vascular endothelial cells were actively involved in the pathophysiological processes of body. The cells of the vascular endothelial cells, are rather active multifunctional team players that could mutually interact with neighboring and circulating cells in complex disease condition. However, there have been few reports exploring the development of diseases from the perspective of the vascular endothelial niche. In the past, a variety of techniques and models have been used to explore the influence of the vascular endothelium on tissues and their related diseases. Using the co-culture system, isolated endothelial cells or blood vessels were found to affect the proliferation and differentiation of other cells and tissues (Shen et al., 2004; Wurmser et al., 2004). Alternatively, vascular endothelial conventional conditional knockdown mice could be used as facilitating devices (Singhal et al., 2021a). Tumor-derived exosomes were additionally added to endothelial cells to investigate their functions in branching and inflammation within the perivascular niche (Rodrigues et al., 2019). Single-cell transcriptomics has been recently applied to trace cell–cell communication within the vascular endothelial niche (Singhal et al., 2021b). Additionally, 3D microfluidic chips have been employed to simulate the microenvironmental characteristics of niches (Zheng et al., 2021).

Vascular endothelial cells are not simply a large group of identical cells, and the phenotypes of endothelial cells exhibit obvious heterogeneity. Vascular endothelial cells from different species and with diverse diameters have disparate phenotypes with respect to their structure, function, and surface molecules. The heterogeneity of endothelial cells is manifested in cell morphology, function, gene expression, antigen synthesis, and niches. The mechanism of heterogeneity might be related to internal factors of cellular gene modification or external factors induced by the extracellular microenvironment. It has been found that the intrinsic factors of endothelial cell heterogeneity are related to the modification of site-specific genes, which regulate the lineage differentiation and formation of endothelial cells. Stolz and Sims-Lucus (Stolz and Sims-Lucas, 2015) believed that endothelial cells in different arteries, or even endothelial cells in different positions within the same artery, exhibiting both partially overlapping and distinct molecular profiles. Chi (Chi et al., 2003) indicated that 14 different loci of arteriovenous endothelial cells and microvascular endothelial cells were found to exhibit heterogeneity in the expression of various genes related to cell function using DNA microarray technology. Microenvironmental factors are external factors regulating the maintenance of endothelial cell specificity, and when the cell microenvironment changes, the cell phenotype also changes accordingly. Microenvironmental factors include both biomechanical signals and biochemical signals. Biomechanical signals comprise shear force and tension of blood flow, while biochemical signals include growth factors, cytokines, hormones, nucleosides, complement, lipoproteins, and extracellular matrix components.

**FIGURE 2:**
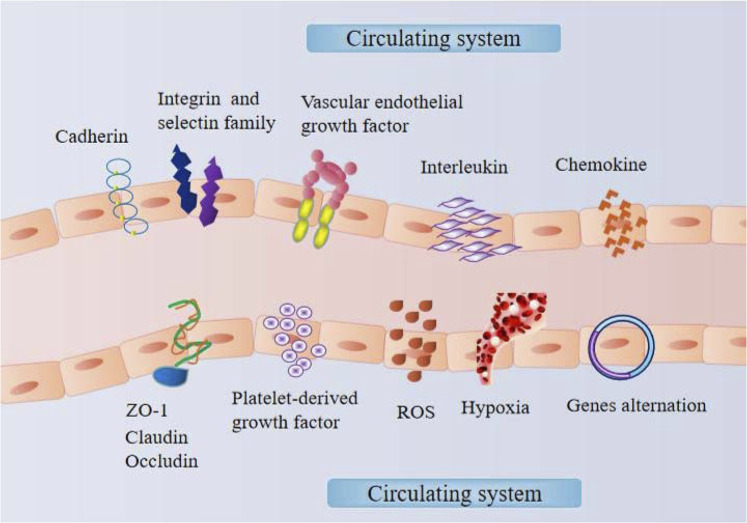
The mechanism within the vascular endothelial niche.

Because vascular tissues are distributed throughout the body, the vascular endothelium acts as the lining of the vascular niche of the body. Vascular endothelial cells can sense and respond to external environmental signals, triggering a series of nonlinear response processes that ultimately lead to posttranscriptional modification or expression changes in genes (Figure 2). With respect to vascular endothelial cells in tissues that cannot be easily obtained, similar cells or tissues could be obtained through heterogeneity stimulation experiments, which is conducive to understanding and diagnosing the pathological mechanisms of various diseases.

## Conclusion

Given that the vascular niche is involved in multiple diseases, biomarkers in the vascular endothelial niche may serve as targets for therapy in various diseases. By predicting biomarkers in the vascular endothelial niche in advance, the threshold of disease intervention could be moved forward, providing targeted prevention and treatment for disease intervention. Vascular endothelial cells might represent a new breakthrough point for diseases, especially drug therapy targeting specific sites of blood vessels, exhibiting good clinical prospect applications.

## References

[B1] Abdel Rahman F. d'Almeida S. Zhang T. Asadi M. Bozoglu T. Bongiovanni D. (2021). Sphingosine-1-Phosphate Attenuates Lipopolysaccharide-Induced Pericyte Loss via Activation of Rho-A and MRTF-A. Thromb. Haemost. 121 (3), 341–350. 10.1055/s-0040-1716844 33011963

[B2] Abkowitz J. L. Robinson A. E. Kale S. Long M. W. Chen J. (2003). Mobilization of Hematopoietic Stem Cells during Homeostasis and after Cytokine Exposure. Blood 102 (4), 1249–1253. 10.1182/blood-2003-01-0318 12714498

[B3] Andersson-Sjöland A. Karlsson J. C. Rydell-Törmänen K. (2016). ROS-induced Endothelial Stress Contributes to Pulmonary Fibrosis through Pericytes and Wnt Signaling. Lab. Invest. 96 (2), 206–217. 10.1038/labinvest.2015.100 26367492

[B4] Beck B. Driessens G. Goossens S. Youssef K. K. Kuchnio A. Caauwe A. (2011). A Vascular Niche and a VEGF-Nrp1 Loop Regulate the Initiation and Stemness of Skin Tumours. Nature 478 (7369), 399–403. 10.1038/nature10525 22012397

[B5] Butler J. M. Kobayashi H. Rafii S. (2010). Instructive Role of the Vascular Niche in Promoting Tumour Growth and Tissue Repair by Angiocrine Factors. Nat. Rev. Cancer 10 (2), 138–146. 10.1038/nrc2791 20094048 PMC2944775

[B6] Cao Z. Ye T. Sun Y. Ji G. Shido K. Chen Y. (2017). Targeting the Vascular and Perivascular Niches as a Regenerative Therapy for Lung and Liver Fibrosis. Sci. Transl. Med. 9 (405), eaai8710. 10.1126/scitranslmed.aai8710 28855398 PMC5606244

[B7] Capel E. Barnier J.-P. Zomer A. L. Bole-Feysot C. Nussbaumer T. Jamet A. (2017). Peripheral Blood Vessels Are a Niche for Blood-Borne Meningococci. Virulence 8 (8), 1808–1819. 10.1080/21505594.2017.1391446 29099305 PMC5810509

[B8] Caporarello N. D’Angeli F. Cambria M. T. Candido S. Giallongo C. Salmeri M. (2019). Pericytes in Microvessels: From "Mural" Function to Brain and Retina Regeneration. Ijms 20 (24), 6351. 10.3390/ijms20246351 PMC6940987 31861092

[B9] Charles N. Ozawa T. Squatrito M. Bleau A.-M. Brennan C. W. Hambardzumyan D. (2010). Perivascular Nitric Oxide Activates Notch Signaling and Promotes Stem-like Character in PDGF-Induced Glioma Cells. Cell Stem Cell 6 (2), 141–152. 10.1016/j.stem.2010.01.001 20144787 PMC3818090

[B10] Chen J. Lippo L. Labella R. Tan S. L. Marsden B. D. Dustin M. L. (2021a). Decreased Blood Vessel Density and Endothelial Cell Subset Dynamics during Ageing of the Endocrine System. EMBO J. 40 (1), e105242. 10.15252/embj.2020105242 33215738 PMC7780152

[B11] Chen J. Sivan U. Tan S. L. Lippo L. De Angelis J. Labella R. (2021b). High-resolution 3D Imaging Uncovers Organ-specific Vascular Control of Tissue Aging. Sci. Adv. 7 (6), eabd7819. 10.1126/sciadv.abd7819 33536212 PMC7857692

[B12] Cheng P. Cao T. Zhao X. Lu W. Miao S. Ning F. (2022). Nidogen1-enriched Extracellular Vesicles Accelerate Angiogenesis and Bone Regeneration by Targeting Myosin-10 to Regulate Endothelial Cell Adhesion. Bioactive Mater. 12, 185–197. 10.1016/j.bioactmat.2021.10.021 PMC8897190 35310379

[B13] Chi J.-T. Chang H. Y. Haraldsen G. Jahnsen F. L. Troyanskaya O. G. Chang D. S. (2003). Endothelial Cell Diversity Revealed by Global Expression Profiling. Proc. Natl. Acad. Sci. U.S.A. 100 (19), 10623–10628. 10.1073/pnas.1434429100 12963823 PMC196854

[B14] Cogle C. R. Goldman D. C. Madlambayan G. J. Leon R. P. Al Masri A. Clark H. A. (2014). Functional Integration of Acute Myeloid Leukemia into the Vascular Niche. Leukemia 28 (10), 1978–1987. 10.1038/leu.2014.109 24637335 PMC4167983

[B15] Colín-Castelán D. Phillips-Farfán B. V. Gutiérrez-Ospina G. Fuentes-Farias A. L. Báez-Saldaña A. Padilla-Cortés P. (2011). EphB4 Is Developmentally and Differentially Regulated in Blood Vessels throughout the Forebrain Neurogenic Niche in the Mouse Brain: Implications for Vascular Remodeling. Brain Res. 1383, 90–98. 10.1016/j.brainres.2011.01.110 21303665

[B16] Czabanka M. Petrilli L. L. Elvers-Hornung S. Bieback K. Albert Imhof B. Vajkoczy P. (2020). Junctional Adhesion Molecule-C Mediates the Recruitment of Embryonic-Endothelial Progenitor Cells to the Perivascular Niche during Tumor Angiogenesis. Ijms 21 (4), 1209. 10.3390/ijms21041209 PMC7072851 32054130

[B17] Ding B.-S. Cao Z. Lis R. Nolan D. J. Guo P. Simons M. (2014). Divergent Angiocrine Signals from Vascular Niche Balance Liver Regeneration and Fibrosis. Nature 505 (7481), 97–102. 10.1038/nature12681 24256728 PMC4142699

[B18] Farahani R. M. Rezaei‐Lotfi S. Simonian M. Xaymardan M. Hunter N. (2019). Neural Microvascular Pericytes Contribute to Human Adult Neurogenesis. J. Comp. Neurol. 527 (4), 780–796. 10.1002/cne.24565 30471080

[B19] Ferentinos P. Tsakirides C. Swainson M. Davison A. Martyn-St James M. Ispoglou T. (2022). The Impact of Different Forms of Exercise on Circulating Endothelial Progenitor Cells in Cardiovascular and Metabolic Disease. Eur. J. Appl. Physiol. 122 (4), 815–860. 10.1007/s00421-021-04876-1 35022875 PMC8927049

[B20] Friedman-Levi Y. Liraz-Zaltsman S. Shemesh C. Rosenblatt K. Kesner E. L. Gincberg G. (2021). Pharmacological Blockers of CCR5 and CXCR4 Improve Recovery after Traumatic Brain Injury. Exp. Neurol. 338, 113604. 10.1016/j.expneurol.2021.113604 33453212

[B21] Géraud C. Koch P.-S. Goerdt S. (2014). Vascular Niches: Endothelial Cells as Tissue- and Site-specific Multifunctional Team Players in Health and Disease. JDDG: J. der Deutschen Dermatologischen Gesellschaft 12 (8), 685–689. 10.1111/ddg.12402 25073555

[B22] Géraud C. Koch P.-S. Zierow J. Klapproth K. Busch K. Olsavszky V. (2017). GATA4-dependent Organ-specific Endothelial Differentiation Controls Liver Development and Embryonic Hematopoiesis. J. Clin. Invest. 127 (3), 1099–1114. 10.1172/JCI90086 28218627 PMC5330741

[B23] Ghajar C. M. Peinado H. Mori H. Matei I. R. Evason K. J. Brazier H. (2013). The Perivascular Niche Regulates Breast Tumour Dormancy. Nat. Cel Biol 15 (7), 807–817. 10.1038/ncb2767 PMC3826912 23728425

[B24] Godavarthy P. S. Kumar R. Herkt S. C. Pereira R. S. Hayduk N. Weissenberger E. S. (2020). The Vascular Bone Marrow Niche Influences Outcome in Chronic Myeloid Leukemia via the E-Selectin - SCL/TAL1 - CD44 axis. Haematologica 105 (1), 136–147. 10.3324/haematol.2018.212365 31018977 PMC6939533

[B25] Grigoryeva E. S. Savelieva O. E. Popova N. O. Cherdyntseva N. V. Perelmuter V. M. (2020). Do tumor Exosome Integrins Alone Determine Organotropic Metastasis? Mol. Biol. Rep. 47 (10), 8145–8157. 10.1007/s11033-020-05826-4 32929649

[B26] Ho I. A. W. Shim W. S. N. (2017). Contribution of the Microenvironmental Niche to Glioblastoma Heterogeneity. Biomed. Res. Int. 2017, 1–13. 10.1155/2017/9634172 PMC5467280 28630875

[B27] Hochstetler C. L. Feng Y. Sacma M. Davis A. K. Rao M. Kuan C.-Y. (2019). KRasG12D Expression in the Bone Marrow Vascular Niche Affects Hematopoiesis with Inflammatory Signals. Exp. Hematol. 79, 3–15. e4. 10.1016/j.exphem.2019.10.003 31669153 PMC6921092

[B28] Hoshizaki K. Takahashi S. Tanaka H. Oki S. Matsushita M. (2022). Stochasticity of Individual Competition and Local Matchup Inequality for Saplings in a Niche‐structured forest. Ecology 103 (4), e3624. 10.1002/ecy.3624 34967952

[B29] Hussain A. Deleuze V. El Kebriti L. Turali H. Pirot N. Glasson Y. (2021). In Lyl1 −/− Mice, Adipose Stem Cell Vascular Niche Impairment Leads to Premature Development of Fat Tissues. Stem Cells 39 (1), 78–91. 10.1002/stem.3286 33022858 PMC7821250

[B30] Infanger D. W. Cho Y. Lopez B. S. Mohanan S. Liu S. C. Gursel D. (2013). Glioblastoma Stem Cells Are Regulated by Interleukin-8 Signaling in a Tumoral Perivascular Niche. Cancer Res. 73 (23), 7079–7089. 10.1158/0008-5472.CAN-13-1355 24121485 PMC3880850

[B31] Jackson W. F. (2022). Endothelial Ion Channels and Cell-Cell Communication in the Microcirculation. Front. Physiol. 13, 805149. 10.3389/fphys.2022.805149 35211031 PMC8861442

[B32] Jing J. Jiang H. Zhang L. (2022). Endothelial Progenitor Cells Promote Neural Stem Cell Proliferation in Hypoxic Conditions through VEGF via the PI3K/AKT Pathway. J. Receptors Signal Transduction 2022, 1–7. 10.1080/10799893.2021.2019275 35042445

[B33] Katsimpardi L. Litterman N. K. Schein P. A. Miller C. M. Loffredo F. S. Wojtkiewicz G. R. (2014). Vascular and Neurogenic Rejuvenation of the Aging Mouse Brain by Young Systemic Factors. Science 344 (6184), 630–634. 10.1126/science.1251141 24797482 PMC4123747

[B34] Kaushansky K. Zhan H. (2018). The Regulation of normal and Neoplastic Hematopoiesis Is Dependent on Microenvironmental Cells. Adv. Biol. Regul. 69, 11–15. 10.1016/j.jbior.2018.06.003 29970351 PMC6102082

[B35] Kopp H.-G. Avecilla S. T. Hooper A. T. Rafii S. (2005). The Bone Marrow Vascular Niche: Home of HSC Differentiation and Mobilization. Physiology 20, 349–356. 10.1152/physiol.00025.2005 16174874

[B36] Kumar N. Saraber P. Ding Z. Kusumbe A. P. (2021). Diversity of Vascular Niches in Bones and Joints during Homeostasis, Ageing, and Diseases. Front. Immunol. 12, 798211. 10.3389/fimmu.2021.798211 34975909 PMC8718446

[B37] Kusumbe A. P. Ramasamy S. K. Itkin T. Mäe M. A. Langen U. H. Betsholtz C. (2016). Age-dependent Modulation of Vascular Niches for Haematopoietic Stem Cells. Nature 532 (7599), 380–384. 10.1038/nature17638 27074508 PMC5035541

[B38] Lai C.-Y. Schwartz B. E. Hsu M.-Y. (2012). CD133+ Melanoma Subpopulations Contribute to Perivascular Niche Morphogenesis and Tumorigenicity through Vasculogenic Mimicry. Cancer Res. 72 (19), 5111–5118. 10.1158/0008-5472.CAN-12-0624 22865455 PMC3463654

[B39] Li L. Man J. Zhao L. (2021). Hypoxia-CXCL6 axis Affects Arteriolar Niche Remodeling in Acute Myeloid Leukemia. Exp. Biol. Med. (Maywood) 246 (1), 84–96. 10.1177/1535370220960675 33167688 PMC7797991

[B40] Li L. Xie T. (2005). Stem Cell Niche: Structure and Function. Annu. Rev. Cel Dev. Biol. 21, 605–631. 10.1146/annurev.cellbio.21.012704.131525 16212509

[B41] Li Q. Ford M. C. Lavik E. B. Madri J. A. (2006). Modeling the Neurovascular Niche: VEGF- and BDNF-Mediated Cross-Talk between Neural Stem Cells and Endothelial Cells: an *In Vitro* Study. J. Neurosci. Res. 84 (8), 1656–1668. 10.1002/jnr.21087 17061253

[B42] Li Q. Zong Y. Li K. Jie X. Hong J. Zhou X. (2019). Involvement of Endothelial CK2 in the Radiation Induced Perivascular Resistant Niche (PVRN) and the Induction of Radioresistance for Non-small Cell Lung Cancer (NSCLC) Cells. Biol. Res. 52 (1), 22. 10.1186/s40659-019-0231-x 30992075 PMC6466699

[B43] Li W. Terada Y. Tyurina Y. Y. Tyurin V. A. Bery A. I. Gauthier J. M. (2022). Necroptosis Triggers Spatially Restricted Neutrophil-Mediated Vascular Damage during Lung Ischemia Reperfusion Injury. Proc. Natl. Acad. Sci. U.S.A. 119 (10), e2111537119. 10.1073/pnas.2111537119 35238643 PMC8917381

[B44] Li Y. Zhang P. Liu Y. Liu W. Yin N. (2016). Helium Preconditioning Protects the Brain against Hypoxia/ischemia Injury via Improving the Neurovascular Niche in a Neonatal Rat Model. Behav. Brain Res. 314, 165–172. 10.1016/j.bbr.2016.08.015 27515290

[B45] Lin C. H. S. Zhang Y. Kaushansky K. Zhan H. (2018). JAK2V617F-bearing Vascular Niche Enhances Malignant Hematopoietic Regeneration Following Radiation Injury. Haematologica 103 (7), 1160–1168. 10.3324/haematol.2017.185736 29567773 PMC6029534

[B46] Lo A. Y. Lai A. W. Ng T. Hung V. L. Tam K. Cheung C. S. (2022). Exacerbated VEGF Up-Regulation Accompanies Diabetes-Aggravated Hemorrhage in Mice after Experimental Cerebral Ischemia and Delayed Reperfusion. Neural Regen. Res. 17 (7), 1566–1575. 10.4103/1673-5374.330612 34916442 PMC8771109

[B47] Lui K. (2014). VEGF-A: the Inductive Angiogenic Factor for Development, Regeneration and Function of Pancreatic Beta Cells. Cscr 9 (5), 396–400. 10.2174/1574888x09666140710100603 25012744

[B48] Luo L.-l. Han J.-x. Wu S.-r. Kasim V. (2022). Intramuscular Injection of Sotagliflozin Promotes Neovascularization in Diabetic Mice through Enhancing Skeletal Muscle Cells Paracrine Function. Acta Pharmacol. Sin. Online ahead of print. 10.1038/s41401-022-00889-4 PMC9525294 35292769

[B49] Lv Y. Fu L. (2018). The Potential Mechanism for Hydroxysafflor Yellow A Attenuating Blood-Brain Barrier Dysfunction via Tight junction Signaling Pathways Excavated by an Integrated Serial Affinity Chromatography and Shotgun Proteomics Analysis Approach. Neurochem. Int. 112, 38–48. 10.1016/j.neuint.2017.10.012 29107696

[B50] Madri J. A. (2009). Modeling the Neurovascular Niche: Implications for Recovery from CNS Injury. J. Physiol. Pharmacol. 60 (Suppl. 4), 95–104. 10.1013/2009/51441418 20083857

[B51] Mammoto A. Mammoto T. (2019). Vascular Niche in Lung Alveolar Development, Homeostasis, and Regeneration. Front. Bioeng. Biotechnol. 7, 318. 10.3389/fbioe.2019.00318 31781555 PMC6861452

[B52] Marchio P. Guerra-Ojeda S. Vila J. M. Aldasoro M. Victor V. M. Mauricio M. D. (2019). Targeting Early Atherosclerosis: a Focus on Oxidative Stress and Inflammation. Oxid Med. Cel Longev 2019, 8563845. 10.1155/2019/8563845 PMC6636482 31354915

[B53] Miwa Y. Hayashi T. Suzuki S. Abe S. Onishi I. Kirimura S. (2013). Up-regulated Expression of CXCL12 in Human Spleens with Extramedullary Haematopoiesis. Pathology 45 (4), 408–416. 10.1097/PAT.0b013e3283613dbf 23619587

[B54] Morita K. Tokushige C. Maeda S. Kiyose H. Noura M. Iwai A. (2018). RUNX Transcription Factors Potentially Control E-Selectin Expression in the Bone Marrow Vascular Niche in Mice. Blood Adv. 2 (5), 509–515. 10.1182/bloodadvances.2017009324 29500219 PMC5851413

[B55] Nambirajan A. Sharma M. C. Gupta R. K. Suri V. Singh M. Sarkar C. (2014). Study of Stem Cell Marker Nestin and its Correlation with Vascular Endothelial Growth Factor and Microvascular Density in Ependymomas. Neuropathol. Appl. Neurobiol. 40 (6), 714–725. 10.1111/nan.12097 24224478

[B56] Nguyen T. S. Lapidot T. Ruf W. (2018). Extravascular Coagulation in Hematopoietic Stem and Progenitor Cell Regulation. Blood 132 (2), 123–131. 10.1182/blood-2017-12-768986 29866813 PMC6634957

[B57] Nikolova G. Strilic B. Lammert E. (2007). The Vascular Niche and its Basement Membrane. Trends Cel Biol. 17 (1), 19–25. 10.1016/j.tcb.2006.11.005 17129728

[B58] Oh M. Zhang Z. Mantesso A. Oklejas A. E. Nör J. E. (2020). Endothelial-Initiated Crosstalk Regulates Dental Pulp Stem Cell Self-Renewal. J. Dent Res. 99 (9), 1102–1111. 10.1177/0022034520925417 32471313 PMC7375737

[B59] Ohab J. J. Fleming S. Blesch A. Carmichael S. T. (2006). A Neurovascular Niche for Neurogenesis after Stroke. J. Neurosci. 26 (50), 13007–13016. 10.1523/JNEUROSCI.4323-06.2006 17167090 PMC6674957

[B60] Olerud J. Johansson Å. Carlsson P.-O. (2009). Vascular Niche of Pancreatic Islets. Expert Rev. Endocrinol. Metab. 4 (5), 481–491. 10.1586/eem.09.25 30736187

[B61] Owen-Woods C. Kusumbe A. (2022). Fundamentals of Bone Vasculature: Specialization, Interactions and Functions. Semin. Cel Develop. Biol. 123, 36–47. 10.1016/j.semcdb.2021.06.025 34281770

[B62] Palikuqi B. Nguyen D.-H. T. Li G. Schreiner R. Pellegata A. F. Liu Y. (2020). Adaptable Haemodynamic Endothelial Cells for Organogenesis and Tumorigenesis. Nature 585 (7825), 426–432. 10.1038/s41586-020-2712-z 32908310 PMC7480005

[B63] Pasquier J. Ghiabi P. Chouchane L. Razzouk K. Rafii S. Rafii A. (2020). Angiocrine Endothelium: from Physiology to Cancer. J. Transl Med. 18 (1), 52. 10.1186/s12967-020-02244-9 32014047 PMC6998193

[B64] Pitt L. A. Tikhonova A. N. Hu H. Trimarchi T. King B. Gong Y. (2015). CXCL12-Producing Vascular Endothelial Niches Control Acute T Cell Leukemia Maintenance. Cancer Cell 27 (6), 755–768. 10.1016/j.ccell.2015.05.0020 26058075 PMC4461838

[B65] Poller W. C. Nahrendorf M. Swirski F. K. (2020). Hematopoiesis and Cardiovascular Disease. Circ. Res. 126 (8), 1061–1085. 10.1161/CIRCRESAHA.120.315895 32271679 PMC7153537

[B66] Poulos M. G. Gars E. J. Gutkin M. C. Kloss C. C. Ginsberg M. Scandura J. M. (2014). Activation of the Vascular Niche Supports Leukemic Progression and Resistance to Chemotherapy. Exp. Hematol. 42 (11), 976–986. 10.1016/j.exphem.2014.08.003 25179751 PMC4254082

[B67] Psaltis P. J. Harbuzariu A. Delacroix S. Holroyd E. W. Simari R. D. (2011). Resident Vascular Progenitor Cells-Diverse Origins, Phenotype, and Function. J. Cardiovasc. Trans. Res. 4 (2), 161–176. 10.1007/s12265-010-9248-9 PMC3048921 21116882

[B68] Qian C. Schoemaker R. G. van Gilst W. H. Yu B. Roks A. J. M. (2008). Regenerative Cell Therapy and Pharmacotherapeutic Intervention in Heart Failure. Nhjl 16 (9), 305–309. 10.1007/BF03086169 PMC2553156 18827874

[B69] Rafii S. Butler J. M. Ding B.-S. (2016). Angiocrine Functions of Organ-specific Endothelial Cells. Nature 529 (7586), 316–325. 10.1038/nature17040 26791722 PMC4878406

[B70] Ribatti D. Basile A. Ruggieri S. Vacca A. (2014). Bone Marrow Vascular Niche and the Control of Angiogenesis in Multiple Myeloma. Front. Biosci. 19, 304–311. 10.2741/4209 24389186

[B71] Ribatti D. Tamma R. Annese T. (2021). The Role of Vascular Niche and Endothelial Cells in Organogenesis and Regeneration. Exp. Cel Res. 398 (1), 112398. 10.1016/j.yexcr.2020.112398 33271129

[B72] Rodrigues G. Hoshino A. Kenific C. M. Matei I. R. Steiner L. Freitas D. (2019). Tumour Exosomal CEMIP Protein Promotes Cancer Cell Colonization in Brain Metastasis. Nat. Cel Biol 21 (11), 1403–1412. 10.1038/s41556-019-0404-4 PMC7354005 31685984

[B73] Rubalcaba J. G. Verberk W. C. E. P. Hendriks A. J. Saris B. Woods H. A. (2020). Oxygen Limitation May Affect the Temperature and Size Dependence of Metabolism in Aquatic Ectotherms. Proc. Natl. Acad. Sci. U.S.A. 117 (50), 31963–31968. 10.1073/pnas.2003292117 33257544 PMC7749359

[B74] Saçma M. Pospiech J. Bogeska R. de Back W. Mallm J.-P. Sakk V. (2019). Haematopoietic Stem Cells in Perisinusoidal Niches Are Protected from Ageing. Nat. Cel Biol 21 (11), 1309–1320. 10.1038/s41556-019-0418-y 31685996

[B75] Sasine J. P. Yeo K. T. Chute J. P. (2017). Concise Review: Paracrine Functions of Vascular Niche Cells in Regulating Hematopoietic Stem Cell Fate. Stem Cell Transl Med 6 (2), 482–489. 10.5966/sctm.2016-0254 PMC5442811 28191767

[B76] Sato Y. Uchida Y. Hu J. Young-Pearse T. L. Niikura T. Mukouyama Y.-s. (2017). Soluble APP Functions as a Vascular Niche Signal that Controls Adult Neural Stem Cell Number. Development 144 (15), 2730–2736. 10.1242/dev.143370 28694255 PMC5560038

[B77] Schiffer D. Annovazzi L. Casalone C. Corona C. Mellai M. (2018). Glioblastoma: Microenvironment and Niche Concept. Cancers 11 (1), 5. 10.3390/cancers11010005 PMC6357107 30577488

[B78] Sharma A. Shiras A. (2016). Cancer Stem Cell-Vascular Endothelial Cell Interactions in Glioblastoma. Biochem. Biophysical Res. Commun. 473 (3), 688–692. 10.1016/j.bbrc.2015.12.022 26692486

[B79] Shen Q. Goderie S. K. Jin L. Karanth N. Sun Y. Abramova N. (2004). Endothelial Cells Stimulate Self-Renewal and Expand Neurogenesis of Neural Stem Cells. Science 304 (5675), 1338–1340. 10.1126/science.1095505 15060285

[B80] Shido K. Chavez D. Cao Z. Ko J. L. Rafii S. Ding B.-S. (2017). Platelets Prime Hematopoietic-Vascular Niche to Drive Angiocrine-Mediated Liver Regeneration. Sig Transduct Target. Ther. 2, 16044. 10.1038/sigtrans.2016.44 PMC5661617 29201496

[B81] Si S. Nakajima-Takagi Y. Iga T. Tsuji M. Hou L. Oshima M. (2018). Hematopoietic Insults Damage Bone Marrow Niche by Activating P53 in Vascular Endothelial Cells. Exp. Hematol. 63, 41–51. 10.1016/j.exphem.2018.04.006 29709619

[B82] Singh A. Veeriah V. Xi P. Labella R. Chen J. Romeo S. G. (2019). Angiocrine Signals Regulate Quiescence and Therapy Resistance in Bone Metastasis. JCI Insight 4 (13), e125679. 10.1172/jci.insight.125679 PMC6629249 31292293

[B83] Singhal M. Augustin H. G. (2020). Beyond Angiogenesis: Exploiting Angiocrine Factors to Restrict Tumor Progression and Metastasis. Cancer Res. 80 (4), 659–662. 10.1158/0008-5472.CAN-19-3351 31831463

[B84] Singhal M. Gengenbacher N. Abdul Pari A. A. Kamiyama M. Hai L. Kuhn B. J. (2021b). Temporal Multi-Omics Identifies LRG1 as a Vascular Niche Instructor of Metastasis. Sci. Transl Med. 13 (609), eabe6805. 10.1126/scitranslmed.abe6805 34516824 PMC7614902

[B85] Singhal M. Gengenbacher N. Abdul Pari A. A. Kamiyama M. Hai L. Kuhn B. J. (2021a). Temporal Multi-Omics Identifies LRG1 as a Vascular Niche Instructor of Metastasis. Sci. Transl. Med. 13 (609), eabe6805. 10.1126/scitranslmed.abe6805 34516824 PMC7614902

[B86] Stolz D. B. Sims-Lucas S. (2015). Unwrapping the Origins and Roles of the Renal Endothelium. Pediatr. Nephrol. 30 (6), 865–872. 10.1007/s00467-014-2798-3 24633402 PMC4164630

[B87] Tanaka M. Koyama T. Sakurai T. Kamiyoshi A. Ichikawa-Shindo Y. Kawate H. (2016). The Endothelial Adrenomedullin-RAMP2 System Regulates Vascular Integrity and Suppresses Tumour Metastasis. Cardiovasc. Res. 111 (4), 398–409. 10.1093/cvr/cvw166 27307317

[B88] Tracy E. P. Stielberg V. Rowe G. Benson D. Nunes S. S. Hoying J. B. (2022). State of the Field: Cellular and Exosomal Therapeutic Approaches in Vascular Regeneration. Am. J. Physiol. Heart Circ. Physiol. 322 (4), H647–H680. 10.1152/ajpheart.00674.2021 35179976 PMC8957327

[B89] Tsai T. L. Wang B. Squire M. W. Guo L. W. Li W. J. (2015). Endothelial Cells Direct Human Mesenchymal Stem Cells for Osteo- and Chondro-Lineage Differentiation through Endothelin-1 and AKT Signaling. Stem Cel Res Ther 6 (1), 88. 10.1186/s13287-015-0065-6 PMC4416238 25998005

[B90] Tung H. H. Lee S. L. (2017). Physical Binding of Endothelial MCAM and Neural Transmembrane Protease Matriptase-Novel Cell Adhesion in Neural Stem Cell Vascular Niche. Sci. Rep. 7 (1), 4946. 10.1038/s41598-017-05131-4 28694515 PMC5504030

[B91] Turpin A. Sharif A. Stoven L. Blond S. Maurage C. A. Le Rhun É. (2015). La niche des cellules souches tumorales dans le glioblastome : des aspects fondamentaux au ciblage thérapeutique [The stem cell niche in glioblastoma: from fundamental aspects to targeted therapies]. Bull. Cancer 102 (1), 24–33. 10.1016/j.bulcan.2014.07.001 25609493

[B92] Willert K. Brown J. D. Danenberg E. Duncan A. W. Weissman I. L. Reya T. (2003). Wnt Proteins Are Lipid-Modified and Can Act as Stem Cell Growth Factors. Nature 423 (6938), 448–452. 10.1038/nature01611 12717451

[B93] Winkler I. G. Barbier V. Nowlan B. Jacobsen R. N. Forristal C. E. Patton J. T. (2012). Vascular Niche E-Selectin Regulates Hematopoietic Stem Cell Dormancy, Self Renewal and Chemoresistance. Nat. Med. 18 (11), 1651–1657. 10.1038/nm.2969 23086476

[B94] Wu L. Mo W. Zhang Y. Zhou M. Li Y. Zhou R. (2017). Vascular and Perivascular Niches, but Not the Osteoblastic Niche, Are Numerically Restored Following Allogeneic Hematopoietic Stem Cell Transplantation in Patients with Aplastic Anemia. Int. J. Hematol. 106 (1), 71–81. 10.1007/s12185-017-2217-1 28303517

[B95] Wurmser A. E. Nakashima K. Summers R. G. Toni N. D'Amour K. A. Lie D. C. (2004). Cell Fusion-independent Differentiation of Neural Stem Cells to the Endothelial Lineage. Nature 430 (6997), 350–356. 10.1038/nature02604 15254537

[B96] Yamagata K. (2019). Soy Isoflavones Inhibit Endothelial Cell Dysfunction and Prevent Cardiovascular Disease. J. Cardiovasc. Pharmacol. 74 (3), 201–209. 10.1097/FJC.0000000000000708 31356541

[B97] Yan X. Dai X. He L. Ling X. Shao M. Zhang C. (2017). A Novel CXCR4 Antagonist Enhances Angiogenesis via Modifying the Ischaemic Tissue Environment. J. Cel Mol Med 21 (10), 2298–2307. 10.1111/jcmm.13150 PMC5618675 28374486

[B98] Yu D. C. Chen J. Ding Y. T. (2010). Hypoxic and Highly Angiogenic Non-tumor Tissues Surrounding Hepatocellular Carcinoma: the 'niche' of Endothelial Progenitor Cells. Int. J. Mol. Sci. 11 (8), 2901–2909. 10.3390/ijms11082901 21152281 PMC2996747

[B99] Zeng Z. Li Y. Pan Y. Lan X. Song F. Sun J. (2018a). Cancer-derived Exosomal miR-25-3p Promotes Pre-metastatic Niche Formation by Inducing Vascular Permeability and Angiogenesis. Nat. Commun. 9 (1), 5395. 10.1038/s41467-018-07810-w 30568162 PMC6300604

[B100] Zhan H. Lin C. Segal Y. Kaushansky K. (2018b). The JAK2V617F-Bearing Vascular Niche Promotes Clonal Expansion in Myeloproliferative Neoplasms. Leukemia 32 (2), 462–469. 10.1038/leu.2017.233 28744010 PMC5783797

[B101] Zhang C. D. Liu M. Wang X. L. Chen S. Fu X. J. Li G. (2022a). Mechanism of CircANKRD36 Regulating Cell Heterogeneity and Endothelial Mesenchymal Transition in Aortic Valve Stromal Cells by Regulating miR-599 and TGF-β Signaling Pathway. Int. J. Cardiol. 352, 104–114. 10.1016/j.ijcard.2022.01.043 35074490

[B102] Zhang S. Zhao L. Shen L. Xu D. Huang B. Wang Q. (2012). Comparison of Various Niches for Endothelial Progenitor Cell Therapy on Ischemic Myocardial Repair: Coexistence of Host Collateralization and Akt-Mediated Angiogenesis Produces a superior Microenvironment. Arterioscler Thromb. Vasc. Biol. 32 (4), 910–923. 10.1161/ATVBAHA.111.244970 22328781

[B103] Zhang T. Lei M. L. Zhou H. Chen Z. Z. Peng Shi P. (2022b). Phylogenetic Relationships of the Zokor Genus Eospalax (Mammalia, Rodentia, Spalacidae) Inferred from Whole-Genome Analyses, with Description of a New Species Endemic to Hengduan Mountains. Zool Res. 43 (3), 331–342. 10.24272/j.issn.2095-8137.2022.045 35301829 PMC9113976

[B104] Zhang Z. Dong Z. Lauxen I. S. Filho M. S. Nör J. E. (2014). Endothelial Cell-Secreted EGF Induces Epithelial to Mesenchymal Transition and Endows Head and Neck Cancer Cells with Stem-like Phenotype. Cancer Res. 74 (10), 2869–2881. 10.1158/0008-5472.CAN-13-2032 24686166 PMC4028029

[B105] Zheng Z. Q. Chen J. T. Zheng M. C. Yang L. J. Wang J. M. Liu Q. L. (2021). Nestin+/CD31+ Cells in the Hypoxic Perivascular Niche Regulate Glioblastoma Chemoresistance by Upregulating JAG1 and DLL4. Neuro Oncol. 23 (6), 905–919. 10.1093/neuonc/noaa265 33249476 PMC8168822

